# Duty-Cycle Electronically Tunable Triangular/Square Wave Generator Using LT1228 Commercially Available ICs for Capacitive Sensor Interfacing

**DOI:** 10.3390/s22134922

**Published:** 2022-06-29

**Authors:** Phamorn Silapan, Pawich Choykhuntod, Rapeepan Kaewon, Winai Jaikla

**Affiliations:** 1 Department of Electrical Engineering, Faculty of Engineering and Industrial Technology, Sanam Chandra Palace Campus, Silpakorn University, Nakhonpathom 73000, Thailand; choychoykhuntod@gmail.com (P.C.); kaewon_r@su.ac.th (R.K.); 2 Department of Engineering Education, School of Industrial Education and Technology, King Mongkut’s Institute of Technology Ladkrabang, Bangkok 10520, Thailand; winai.ja@kmitl.ac.th

**Keywords:** electronically tunable, triangular/square wave generator, LT1228

## Abstract

This paper proposes a duty-cycle electronically tunable triangular/square wave generator using LT1228 commercially available ICs for capacitive sensor interfacing. The generator comprises two LT1228s, a grounded resistor, and a grounded capacitor. The circuit provides two output signals which are triangular and square waves. Both signals are regulated by adjusting the current bias. Likewise, the amplitude of the triangular signal can be tuned electronically without affecting the frequency. In addition, the square wave can independently control the linear duty cycle via tuning the voltage. Experiment results confirm the performance of the proposed circuit that the amplitude of the triangular wave, frequency, and duty cycle are linearly controllable via current or voltage, which do not affect each other. The duty cycle, the amplitude of the triangular wave, and frequency have maximum errors of ±1.60%, ±3.33%, and ±2.55%, respectively.

## 1. Introduction

**Figure 1:**
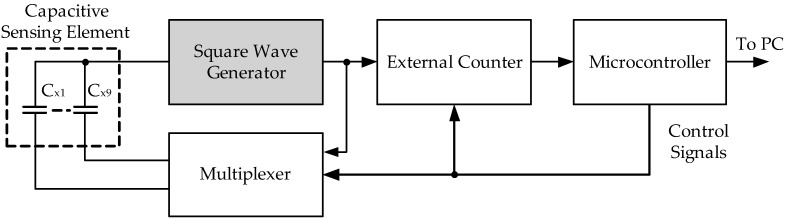
The block diagram of a capacitive sensor interfacing [8].

Many applications use a triangular and square wave generator, such as communication, instrumentation, electrical and control, and electronic systems. In communication systems, a triangular and square wave generator is used to generate carrier signals. Furthermore, a triangular and square shape is applied in generated pulse width modulation (PWM) to control motors, class-D amplifiers, and switching power supplies in electrical, control and electronic systems [1,2,3]. Moreover, it is employed to measure capacitance and capacitive sensor interfacing in instrumentation systems [4,5,6,7,8], as shown in Figure 1.

From capacitive sensor interfacing in [8], it is found that a square wave generator was its main component. There are also a fast counter, a multiplexer, and a microcontroller. The sensing element’s capacitance values are converted to a period modulated by the square wave generator. The counter counts the number of periods that have passed from the beginning of the counter. The measured capacitance is selected by the multiplexer. The microcontroller controls the external counter and multiplexer, which reads the data and sends it to the PC through a data line interface.

Many triangular and square wave generators have been proposed in the literature. Regarding technique design, these circuits are based on a Schmitt trigger with lossy and lossless integrators [9,10,11,12,13,14,15]. The generator in [9] is a low-power circuit and works in the MHz range. However, it consists of a Schmitt trigger with a lossy integrator that provides only a square wave. In addition, the frequency cannot be tuned by the electronic method, and the duty cycle cannot be adjusted. The construction of circuits in [10,11,12,13,14,15] uses the Schmitt trigger cascade, a lossless integrator where the resulting outputs are a triangular and square signal. A generator for instrumentation applications is proposed in [10]. It is a low power circuit, tuning amplitude and frequency by current biases; however, it cannot control the duty cycle. Additionally, the frequency control is non-linear. Thus, it is not easy to control. In 2011 and 2016, square wave generators using OTAs and VDTA were presented, respectively [11,12]. The advantages of these circuits include linearly controlling amplitude and frequency via voltage. However, the duty cycle of [11,12] lacks of electronic control. A CCI- and OTA-based triangular and square wave generator was presented in 2019 [3]. This generator has features such as linear electronic controllability of the frequency. However, it lacks adjustability amplitude and duty cycle of the output signals. Using a Dual-X current conveyor transconductance amplifier (MO-DXCCTA) to design a triangular/square wave generator, which can be implemented by commercially available ICs (AD844 and LM13700) [13], it uses current bias to linearly control frequency, amplitude, and duty cycle. Notwithstanding, controlling the frequency by current bias affects the duty cycle. Then, the frequency can be adjusted independently by adjusting the capacitor, which is difficult to control and non-linear. A dual-mode triangular/square wave generator is implemented by three current feedback amplifiers (CFOAs) [14]. The CFOAs employ commercial ICs, AD844. This circuit can operate for both current and voltage modes. It also gives sawtooth, triangular, and square waves, which can electronically tune the duty cycle, but the generator cannot adjust the frequency and amplitude. In particular, the adjustable frequency with a resistor is non-linear, which makes it difficult to apply in an automatic system.

In the field of analog signal processing, the use of analog function blocks (ABB) for the synthesis and design of high-performance circuits has received considerable attention [15,16,17,18,19,20]. The topologies of analog circuits synthesized from analog function blocks are simple, with a few active and passive elements. The synthesis procedures of circuits using analog function blocks are also easier and more flexible than using transistor levels. With the high input and low out impedance properties of voltage-mode ABB, as well as the low input and high output impedance features of current-mode ABB, additional buffer devices at input and output nodes of some ABB-based circuits are not required. In-circuit design for specific applications, using the commercially available ABB, is more convenient and cheaper than the CMOS or BJT-based ABBs. Although the CMOS-ABB-based circuits provide high circuit performances with small size, low voltage, and low power, the cost is still high for monolithic integrated circuit implementation. Therefore, the prospect of analog circuits using commercially available ABB is frequently proposed [21,22,23,24,25,26]. LT1228 is a popular commercially available ABB. It is the combination of the operational transconductance amplifier (OTA) and current feedback amplifier (CFA). This IC has several advantageous features such as electronic controllability, wide bandwidth, high voltage input impedance, high current output impedance, low voltage output impedance, and a wide range of applications, etc. With these advantageous features, the LT1228-based analog circuits can be found in the open literature [27,28,29,30,31].

From the previous, this research aims to synthesize a triangular/square signal generator employing LT1228 commercially available ICs, which are linearly tunable in amplitude, frequency, and duty cycle, using an electronic method. The paper is classified into six sections. The first section is the introduction. The ideal concept of synthesizing a duty cycle adjustable triangular/square wave generator is expanded in Section 2. The essential operation and saturation mode of LT1228 are described in Section 3. Section 4 clarifies the notion of Schmitt trigger and triangular/square wave generator. Section 5 concerns the performance of the circuit, which is confirmed by the experimental results. Finally, the conclusion is in Section 6.

## 2. Idea of Synthesize of Duty Cycle Adjustable Triangular/Square Wave Generator

**Figure 2:**
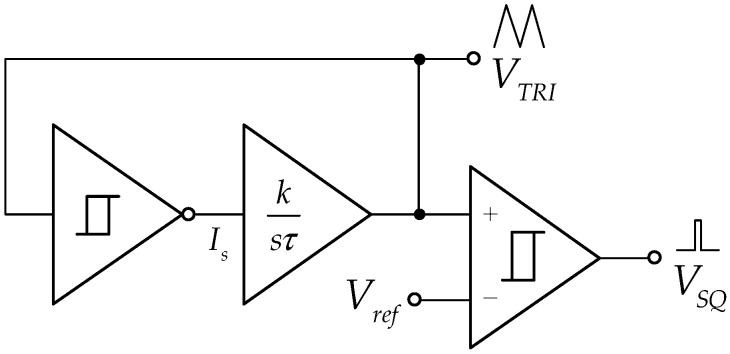
The block diagram of duty cycle adjustable triangular/square wave generator.

Synthesizing the block diagram is shown in Figure 2. It aims to create a triangular/square wave generator that electronically controls the square wave’s duty cycle without affecting amplitude and generated frequency. The cycle adjustable triangular/square wave generator consists of a current-mode inverting Schmitt trigger, a lossless integrator, an inverting amplifier, and a comparator. The output frequency can be found to be

(1) $$f_{o}=\frac{k\left|I_{S}\right|}{4I_{HL}\tau },$$

Where k, $I_{S}$, $I_{TH}$ and τ are gain of the amplifier, the output amplitude of the Schmitt trigger, the current hysteresis of the Schmitt trigger, and the time constant of the lossless integrator. From (1), the frequency control of the system $\left(f_{o}\right)$ can be tuned by k or $I_{HL}$. The triangular wave amplitude can be calculated below:

(2) $$\left|V_{TRI}\right|=k\left|I_{S}\right|,$$

The duty cycle of the triangular/square wave generator can be expressed as

(3) $$D=\frac{1}{2}\left(1-\frac{V_{ref}}{\left|V_{TRI}\right|}\right)\times 100\%,$$

where $V_{ref}$ is the reference voltage. It can be concluded that the duty cycle can be adjusted by $V_{ref}$.

## 3. Off-the-Shelf IC (LT1228)

**Figure 3:**
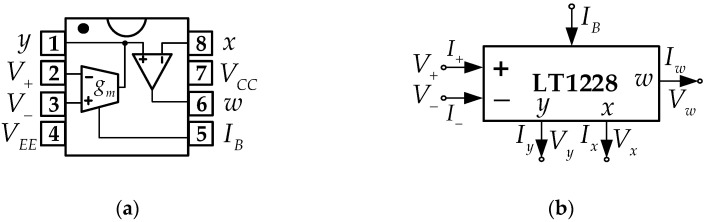
LT1228 (**a**) The connecting of the OTA and CFA in LT1228 [32] (**b**) Symbol.

The LT1228 is an off-the-shelf IC using BJT technology. It implements the current-gain control with an operational transconductance amplifier (Voltage Differencing to Current), whose gain is a direct variation to an externally bias current. The output current is converted to a voltage by an external resistor. The CFA (Current Feedback Amplifier) amplifies the voltage into an 8 port, as shown in Figure 3.

Testing LT1228, it is found that the OTA’s output current ($I_{y}$) is given by,

(4) $$I_{y}=I_{B}\tanh\left(\frac{V_{+}-V_{-}}{3.87V_{T}}\right),$$

where $V_{-}$ and $V_{+}$ are the voltages of pin 2 and 3, respectively. $V_{T}$ is the thermal voltage. $I_{B}$ is the externally bias current of terminal 5. The Maclaurin series of the hyperbolic tangent is as follows [33]:

(5) $$\tanh x=x-\frac{x^{3}}{3}+\frac{2x^{5}}{15}-\frac{17x^{7}}{315}+...,$$

If x≪1, the tanhx term shown in (5) can be estimated as tanhx≈x. From (4), its first-order approximation can be derived to be

(6) $$\tanh\left(\frac{V_{+}-V_{-}}{3.87V_{T}}\right)=\frac{V_{+}-V_{-}}{3.87V_{T}},$$

Therefore, using (6), $I_{y}$ can be rewritten to be

(7) $$I_{y}=\frac{I_{B}{\left(V_{+}-V\right)}_{-}}{3.87V_{T}},$$

it is seen that $I_{y}$ conforms to the datasheet of the LT1228, where $\frac{I_{B}}{3.87V_{T}}$ is a transconductance amplifier $\left(g_{m}\right)$. $V_{T}$ at a temperature of 27 degrees Celsius is about 26 mV. Finally, $I_{y}$ is approximately obtained:

(8) $$I_{y}=10I_{B}\left(V_{+}-V_{-}\right).$$

**Figure 4:**
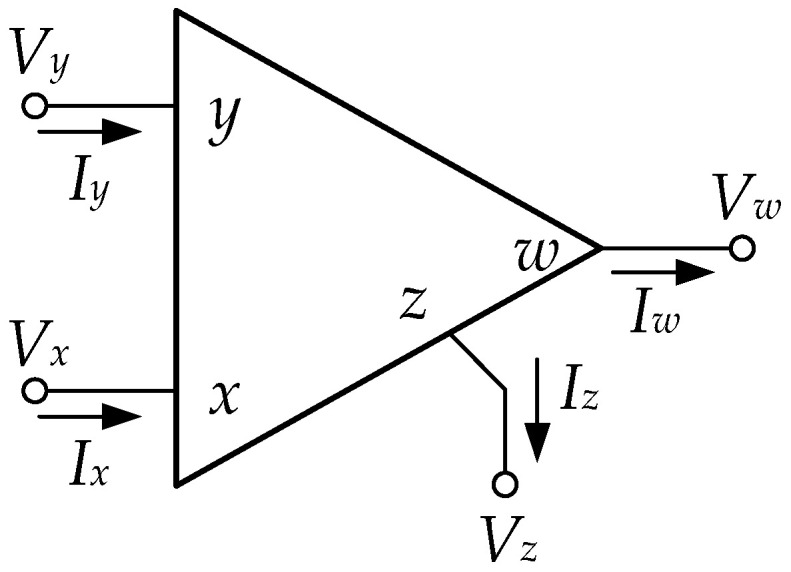
The symbol of General CFA.

From the above, the relationship of voltage and current for each LT1228’s pin is

(9) $$\left[\begin{array}{l} I_{+}\\ I_{-}\\ I_{y}\\ V_{x}\\ V_{w} \end{array}\right]=\left[\begin{array}{ccccc} 0 & 0 & 0 & 0 & 0\\ 0 & 0 & 0 & 0 & 0\\ g_{m} & -g_{m} & 0 & 0 & 0\\ 0 & 0 & 1 & 0 & 0\\ 0 & 0 & 0 & Z_{T} & 0 \end{array}\right]\left[\begin{array}{l} V_{+}\\ V_{-}\\ V_{y}\\ I_{x}\\ I_{w} \end{array}\right],$$

where $V_{w}$, $V_{x}$, and $V_{y}$ are the voltage of pin 6, 8, and 1, respectively. $I_{w}$ and $I_{x}$ are the current of pins 6 and 8, respectively. Generally, CFA has 4 terminals: *y*, *x*, *z*, and *w*, as shown in Figure 4. However, the pin z of CFA is inside the LT1228, and this pin is floated. The trans-resistance gain of the pin z is $Z_{T}$, which is ideally about infinity. From the LT1228 test, it is found that $Z_{T}$ is approximately 190 kΩ. Then, (9) can be rewritten as follows:

(10) $$\left[\begin{array}{l} I_{+}\\ I_{-}\\ I_{y}\\ V_{x}\\ V_{w} \end{array}\right]=\left[\begin{array}{ccccc} 0 & 0 & 0 & 0 & 0\\ 0 & 0 & 0 & 0 & 0\\ g_{m} & -g_{m} & 0 & 0 & 0\\ 0 & 0 & 1 & 0 & 0\\ 0 & 0 & 0 & 190\;k & 0 \end{array}\right]\left[\begin{array}{l} V_{+}\\ V_{-}\\ V_{y}\\ I_{x}\\ I_{w} \end{array}\right],$$

## 4. Concept of the Duty Cycle Tunable Triangular/Square Wave Generator

### 4.1. Concept of Schmitt Trigger

Designing the Schmitt trigger uses the LT1228’s saturation mode, which is both internal OTA and CFA. The OTA operation condition in the saturation region is the input voltage differencing $\left(V_{+}-V_{-}\right)$ more than 150 mV or less than −150 mV. Thus, the output current $\left(I_{y}\right)$ can be found in OTA’s saturation region to be

(11) $$I_{y}=\{\begin{array}{c} I_{B}\\ -I_{B} \end{array}\begin{array}{c} if\\ if \end{array}\begin{array}{c} V_{+}-V_{-}\\ V_{+}-V_{-} \end{array}\begin{array}{c} \geq 150\;\mathrm{mV}\\ \leq -150\;\mathrm{mV} \end{array},$$

**Figure 5:**
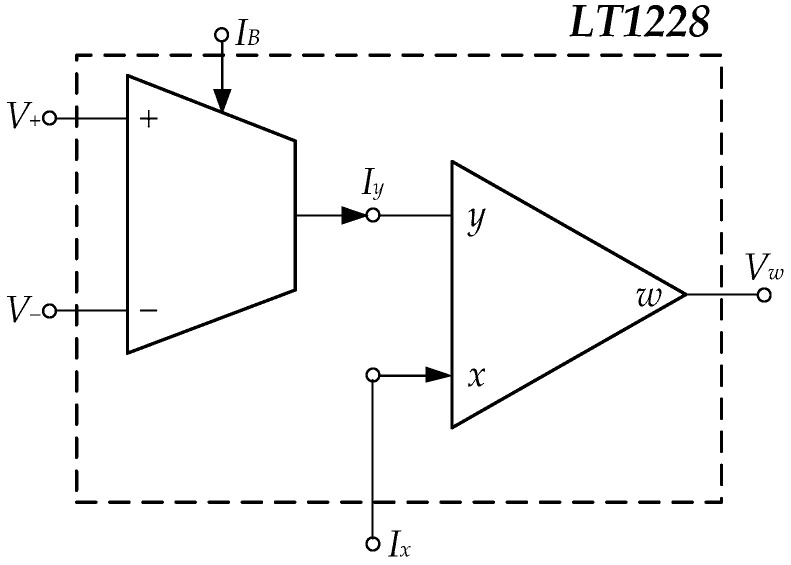
The internal construction of LT1228.

**Figure 6:**
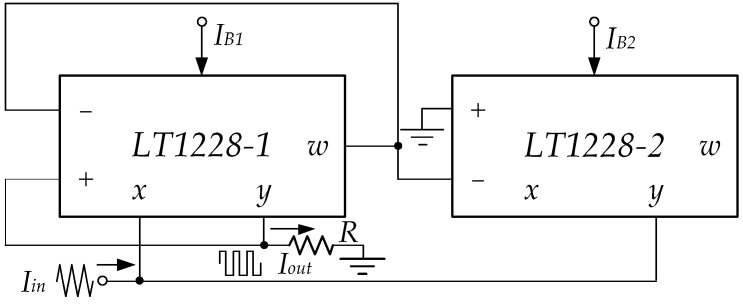
The Schmitt trigger using LT1228s.

From Figure 5, the +, −, and y ports are floated. The CFA operates in saturation mode when $I_{x}\left(190k\right)\geq 0$ or $I_{x}\left(190k\right)\leq 0$. So $V_{w}$ is expressed as

(12) $$V_{w}=\{\begin{array}{c} V_{SAT}\\ -V_{SAT} \end{array}\begin{array}{c} if\\ if \end{array}\begin{array}{c} I_{x}\left(190\;k\right)\geq 0\\ I_{x}\left(190\;k\right)\leq 0 \end{array}=\{\begin{array}{c} V_{CC}\\ V_{EE} \end{array}\begin{array}{c} if\\ if \end{array}\begin{array}{c} I_{x}\left(190\;k\right)\geq 0\\ I_{x}\left(190\;k\right)\leq 0 \end{array},$$

where $V_{SAT}$ and $-V_{SAT}$ are the positive and negative saturation voltages; $V_{CC}$ and $V_{EE}$ are the positive and negative supply voltages, respectively. Using LT1228’s saturation mode, the Schmitt trigger is shown in Figure 6, which consists of two LT1228s and a grounded resistor.

**Figure 7:**
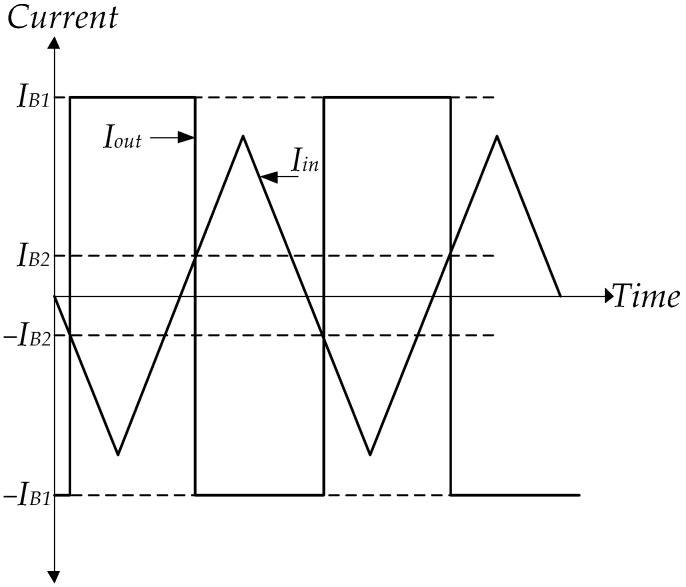
The waveform of Schmitt trigger using LT1228s.

At the initial time, the triangular wave is the input signal of LT1228-1’s pin x, which $V_{w1}$ is about $I_{in}\left(190\;k\right)$. From Figure 6, it is found that the $V_{w1}$ equals $V_{-}$ of LT1228-1 $\left(V_{1-}\right)$ and LT1228-2 $\left(V_{2-}\right)$. Thus, OTAs of LT1228-2 operates in saturation mode; LT1228-2’s $I_{y}\left(I_{y2}\right)$ is $-I_{B2}$. At the same time, $V_{1-}=I_{in}\left(190\;k\right)$, while $V_{1+}=RI_{y1}$. Then, $V_{1+}-V_{1-}$ is much more than |150 mV|, where LT1228-1 is saturation mode operational. OTAs of LT1228-1 and LT1228-2 operate in saturation mode; LT1228-1’s $I_{y}\left(I_{out}\right)$ and LT1228-2’s $I_{y}\left(I_{y2}\right)$ are approximated $-I_{B1}$ and $-I_{B2}$, respectively. $I_{out}$ equals $-I_{B1}$ until $I_{in}$ is lower than $-I_{B2}$. $I_{out}$ is changed from $-I_{B1}$ to $I_{B1}$ while $I_{y2}$ is equal to $I_{B2}$. $I_{out}$ is returned to be $-I_{B1}$ again when $I_{in}$ is higher than $I_{B2}$, as illustrated in Figure 7.

Hence, the output current $\left(I_{out}\right)$ can be obtained by,

(13) $$I_{out}=\{\begin{array}{c} -I_{B1}\\ I_{B1} \end{array}\begin{array}{c} if\\ if \end{array}\begin{array}{c} I_{in}\\ I_{in} \end{array}\begin{array}{c} \geq I_{HL}\\ \leq I_{LH} \end{array},$$

where $I_{HL}$ and $I_{LH}$ are the high and low hysteresis currents, which can be found to be

(14) $$I_{HL}=I_{B2}\;\mathrm{and}\;I_{LH}=-I_{B2}.$$

**Figure 8:**
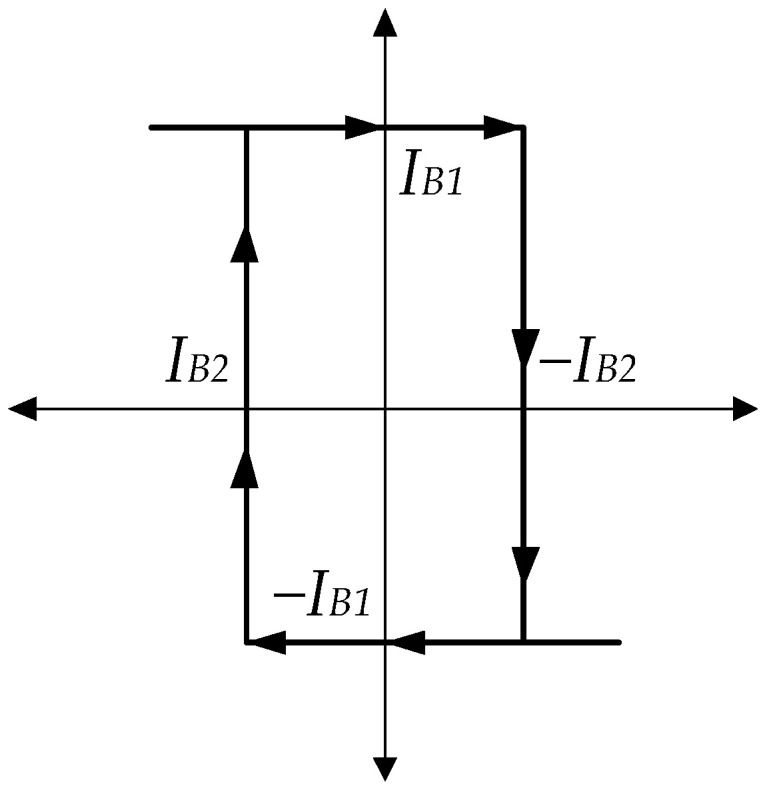
The DC characteristic of the Schmitt trigger using LT1228s.

Using (14), $I_{out}$ can be recalculated to be

(15) $$I_{out}=\{\begin{array}{c} -I_{B1}\\ I_{B1} \end{array}\begin{array}{c} if\\ if \end{array}\begin{array}{c} I_{in}\\ I_{in} \end{array}\begin{array}{c} \geq I_{B2}\\ \leq -I_{B2} \end{array},$$

where $I_{B1}$ and $I_{B2}$ are the external current bias of LT1228-1 and LT1228-2, respectively. The external bias current linearly controls the output and hysteresis currents of the Schmitt trigger, which are $I_{B1}$ and $I_{B2}$. Additionally, they are insensitive to temperature. From (13) and (14), the DC characteristic of the Schmitt trigger is displayed in Figure 8. It is seen that it is a noninverting Schmitt trigger.

### 4.2. Concept of Triangular/Square Wave Generator

**Figure 9:**
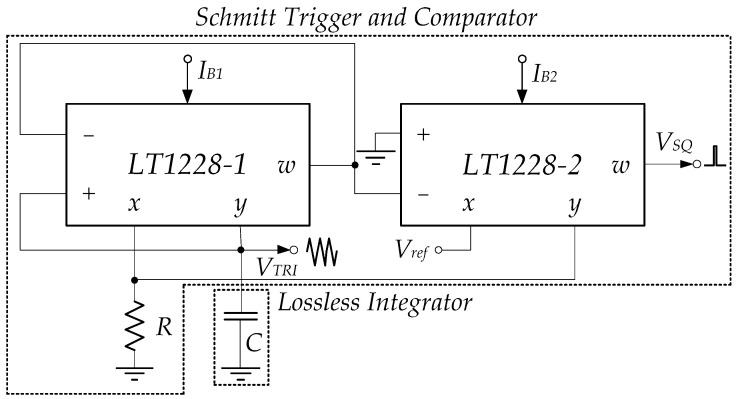
The duty cycle tunable triangular/square wave generator using LT1228s.

The duty cycle tunable triangular/square wave generator using LT1228s is explained in this section. Using the Schmitt trigger in Section 4.1, the capacitor and resistor are replaced by the resistor and the input signal at LT1228-1’s *y* and x terminals, respectively, as shown in Figure 9, which operates as a triangular wave generator.

Using properties of LT1228, $V_{y1}=V_{x1}=V_{TRI}$, thus the amplitude of the triangular wave $\left(V_{TRI}\right)$ can be found to be

(16) $$\left|V_{TRI}\right|=R\left|I_{B2}\right|.$$

**Figure 10:**
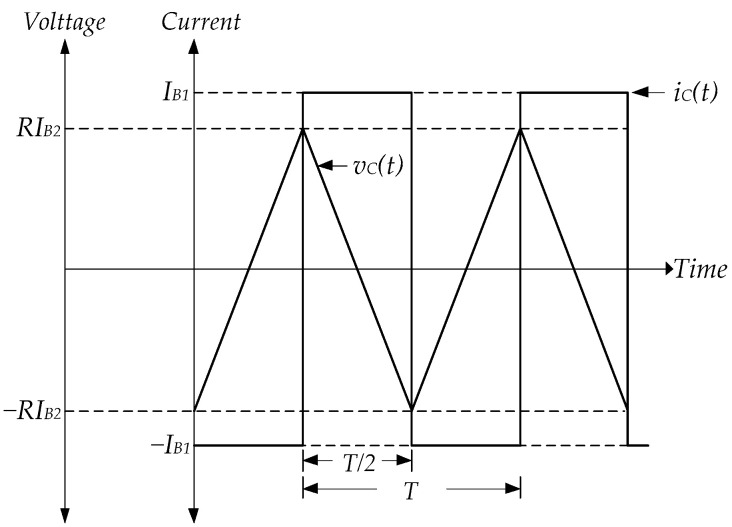
The waveform of the capacitor’s voltage and current using LT1228s.

Using Figure 10, the period of the signal depends on the amplitude of the triangular wave; therefore, it can be expressed as,

(17) $$\frac{T}{2}=\frac{C}{i_{C}}\int_{-v_{C}}^{v_{C}}dv_{C}=\frac{C}{\left|I_{B1}\right|}\int_{-RI_{B2}}^{RI_{B2}}dv_{C},$$

and

(18) $$T=\frac{4RC\left|I_{B2}\right|}{\left|I_{B1}\right|}.$$

From (18), the frequency can be derived as

(19) $$f=\frac{1}{T}=\frac{\left|I_{B1}\right|}{4RC\left|I_{B2}\right|}.$$

Independently controlling the frequency can be archived by the current bias of LT1228-1 $\left(I_{B1}\right).$ In addition, since (19) is without $V_{T}$ term, the frequency is not sensitive to temperature variation. The open-loop voltage gain of LT1228’s internal CFA is high, which is about 55 dB [30], so the LT1228-2’s CFA is used as a voltage comparator. The inputs of the comparator are the DC voltage reference $\left(V_{ref}\right).$ and the triangular signal $\left(V_{TRI}\right).$ The resulting output is a square signal. The output amplitude can be

(20) $$V_{SQ}=V_{w2}=\{\begin{array}{c} V_{SAT}\\ -V_{SAT} \end{array}\begin{array}{c} if\\ if \end{array}\begin{array}{c} V_{TRI}\geq V_{ref}\\ V_{TRI}\leq V_{ref} \end{array}.$$

**Figure 11:**
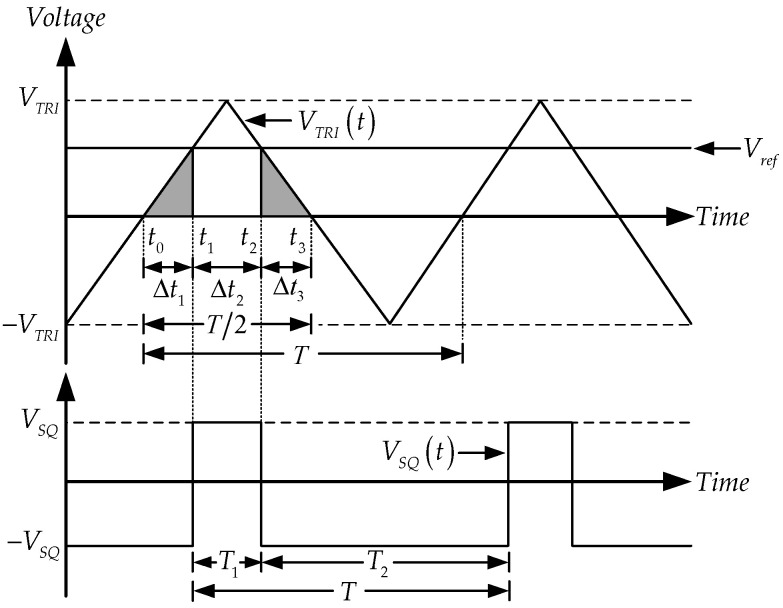
The waveform of the triangular/square wave generator using LT1228s.

As demonstrated in Figure 11, the rise time and fall time depend on $V_{ref}$, and it can be seen that $V_{SQ}$ equals $V_{SAT}$ until $V_{TRI}\left(t\right)$ or $RI_{B2}\left(t\right)$ less than $V_{ref}$. The amplitude of the square wave is changed $V_{SAT}$ to $-V_{SAT}$. It becomes $V_{SAT}$ again when $V_{TRI}\left(t\right)$ is upward $V_{ref}$. From the relationship mentioned above, It is the period when $V_{TRI}\left(t\right)$ goes up and then equals $V_{ref}$ and where $V_{TRI}\left(t\right)$ goes down and is equal to $V_{ref}$, which is the pulse width $\left(\Delta t_{2}\right)$.

It is discovered that $\Delta t_{2}$ or $T_{1}$ is the difference between $\frac{T}{2}$ and $\Delta t_{1}+\Delta t_{3}$; it can be written as follows:

(21) $$\Delta t_{2}=T_{1}=\frac{T}{2}-\Delta t_{1}-\Delta t_{3},$$

where $\Delta t_{1}$ and $\Delta t_{3}$ are the duration times of addition $V_{TRI}\left(t\right)$ from 0 to $V_{ref}$, and the decrease $V_{TRI}\left(t\right)$ from $V_{ref}$ to 0, respectively. Using the straight-line equation, the positive and negative slopes of the triangular signal are given by

(22) m=2VTRIT2=4R|IB2|T and −m=−2VTRIT2=−4R|IB2|T

Therefore, $\Delta t_{1}$ and $\Delta t_{3}$ are obtained:

(23) $$\Delta t_{1}=\frac{V_{TRI}\left(t_{1}\right)-V_{TRI}\left(t_{0}\right)}{m}=\frac{V_{ref}-0}{m}=\frac{V_{ref}}{m},$$

and

(24) $$\Delta t_{3}=\frac{V_{TRI}\left(t_{3}\right)-V_{TRI}\left(t_{2}\right)}{-m}=\frac{0-V_{ref}}{-m}=\frac{V_{ref}}{m}.$$

By Substituting (22) in (23) and (24), $\Delta t_{1}$ and $\Delta t_{1}$ can be rewritten by

(25) $$\Delta t_{1}=\Delta t_{3}=\frac{V_{ref}T}{4R\left|I_{B2}\right|},$$

(24) and (25) can be substituted in (21), $\Delta t_{2}$ can be obtained by

(26) $$\Delta t_{2}=T_{1}=\frac{T}{2}-\frac{V_{ref}T}{4R\left|I_{B2}\right|}=\frac{T}{2}\left(1-\frac{V_{ref}}{2R\left|I_{B2}\right|}\right).$$

Using (18) and (26), the duty cycle (D) is as follows:

(27) $$D=\frac{T_{1}}{T}\times 100\%=\frac{1}{2}\left(1-\frac{V_{ref}}{2R\left|I_{B2}\right|}\right)\times 100\%.$$

From (16), (19), (20) and (26), it is established that the triangular/square wave generator is slightly affected by temperature changes. The current biases have the ability to control the amplitude of the triangular wave and the frequency. Additionally, the frequency can be independently tuned without affecting the triangle wave’s amplitude by $I_{B1}$. $V_{ref}$ can control the duty cycle of the square signal.

### 4.3. Non-Ideal Case of LT1228 on Saturation-Mode

**Figure 12:**
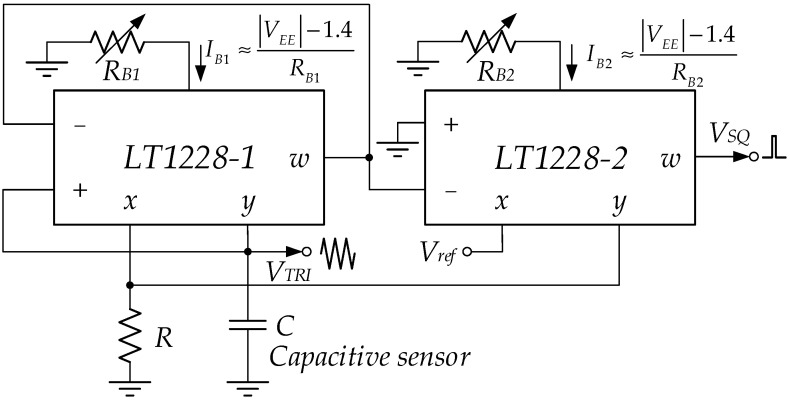
The manual calibration through *R*_*B*1_ or *R*_*B*2_.

**Figure 13:**
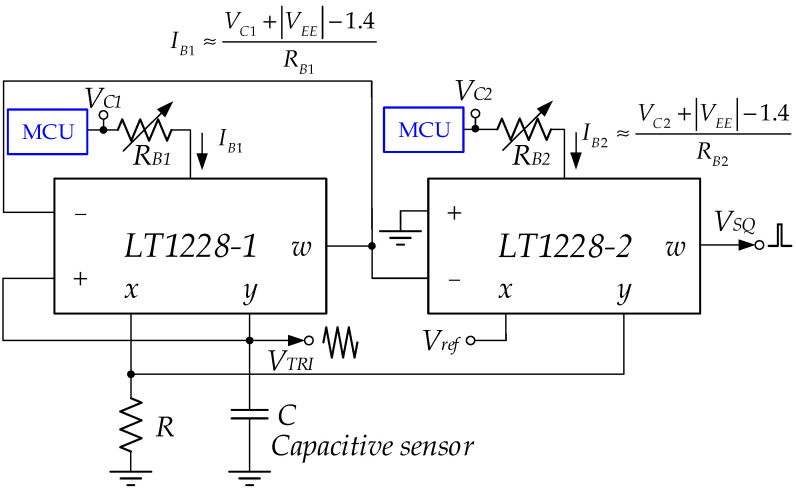
The automatic calibration through *V*_*C*1_ or *V*_*C*2_.

The non-ideal effect of LT1228 in the saturation-mode region on the operation of the proposed triangular/square wave generator is expanded in this part; the current and voltage’s relationship of LT1228 on saturation mode in non-ideal is given as follows:

(28) $$V_{y}=\beta_{x}V_{x},\;I_{y}=\{\begin{array}{c} \alpha I_{B}\\ -\alpha I_{B} \end{array}\begin{array}{c} if\\ if \end{array}\begin{array}{c} V_{+}-V_{-}\\ V_{+}-V_{-} \end{array}\begin{array}{c} \geq 150\;\mathrm{mV}\\ \leq -150\;\mathrm{mV} \end{array},$$

and

(29) $$V_{w}=\{\begin{array}{c} V_{SAT}\\ -V_{SAT} \end{array}\begin{array}{c} if\\ if \end{array}\begin{array}{c} I_{x}\left(190\;k\right)\geq 0\\ I_{x}\left(190\;k\right)\leq 0 \end{array}=\{\begin{array}{c} \beta V_{CC}\\ \beta V_{EE} \end{array}\begin{array}{c} if\\ if \end{array}\begin{array}{c} I_{x}\left(190\;k\right)\geq 0\\ I_{x}\left(190\;k\right)\leq 0 \end{array},$$

where α, $\beta_{x}$, and β are the current error outputs, voltage gain transfer at *x* pin error, and voltage error output, respectively. Taking into account the non-idealities of LT1228 on the saturation region, so the non-ideal amplitude of the triangular and square wave, frequency, and duty cycle can be recalculated by

(30) $$\left|V_{TRI}\right|=\alpha_{2}R\left|I_{B2}\right|,$$

(31) $$V_{SQ}=\{\begin{array}{c} \beta_{2}V_{CC}\\ \beta_{2}V_{EE} \end{array}\begin{array}{c} if\\ if \end{array}\begin{array}{c} V_{TRI}\geq V_{ref}\\ V_{TRI}\leq V_{ref} \end{array},$$

(32) $$f=\frac{1}{T}=\frac{\alpha_{1}\left|I_{B1}\right|}{4\alpha_{2}\beta_{x1}RC\left|I_{B2}\right|},$$

and

(33) $$D=\frac{T_{1}}{T}\times 100\%=\frac{1}{2}\left(1-\frac{V_{ref}}{2\alpha_{2}\beta_{x1}R\left|I_{B2}\right|}\right)\times 100\%,$$

where $\alpha_{1}$ and $\alpha_{2}$ respectively, are the current error outputs of LT1228-1 and LT1228-2; $\beta_{x1}$ is the voltage gain transfer at the x pin error of LT1228-1; $\beta_{2}$ is LT1228-2’s voltage error output. It is found that the imperfections of LT1228 affect how well the proposed triangular/square wave generator works; applied in the capacitive sensor interface by plugging in the capacitive sensor instead of the capacitor. For example, HCH-1000 has a capacitance value of 310 pF to 350 pF [34]. The capacitance of this sensor can overcome the parasitic capacitance at the *Y* and + terminals, which are 6 pF and 3 pF, respectively [35]. For the accurate readout value, it can be calibrated via the bias currents $I_{B1}$ or $I_{B2}$. There are two ways to achieve this: manually and automatically. Manually, it is an $I_{B1}$ or $I_{B2}$ adjustment through the bias resistors, $R_{B1}$ or $R_{B2}$, as shown in Figure 12. Automatically, the bias currents $I_{B1}$ or $I_{B2}$ are tuned via the control voltages $V_{C1}$ and $V_{C2}$, which is a convenient electronic method controlled by the microcontroller unit (MCU), as shown in Figure 13. However, $I_{B1}$ or $I_{B2}$ depend on the temperature and negative power supply voltage ($V_{EE}$).

## 5. Experimental Results

This section shows the experimental results to ensure the efficiency of the triangular/square wave generator using LT1228s. RIGOL DS1054Z (RIGOL Technologies Co. Ltd, Beijing, China) is the measuring instrument that was utilized. In the experiment, $I_{B1}=800\;\mu A$, $I_{B2}=200\;\mu A$, R=1 kΩ, C=0.1 μF, ±9 V supply voltage was used.

**Figure 14:**
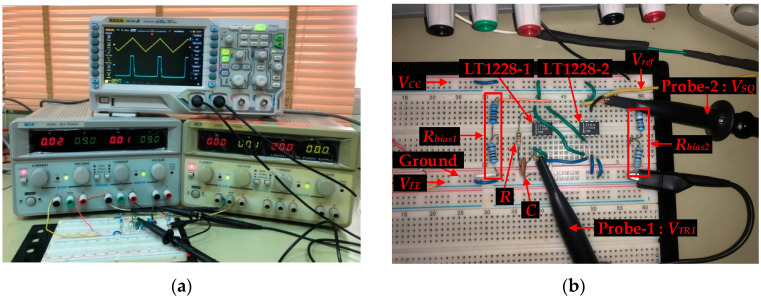
The real implementation of a triangular/square wave generator (**a**) the setup for the experiment (**b**) The actual triangular/square wave generator implementation.

Figure 14a shows the experimental setup, consisting of one RIGOL DS1054, a breadboard, two DC power supplies, and two ×10 oscilloscope probes. The first DC power supply is MCP MODEL: M10-TP3005H which is ±9 V supply voltage. $V_{ref}$ uses MCP MODEL: M10-TP3003L for DC supply, which is set to 168 mV at the moment. The actual triangular/square wave generator implementation is displayed in Figure 14b. It is composed of five main parts: R=1 kΩ, C=0.1 μF, two LT1228s, $R_{bias1}=10.62\;k\Omega$, and $R_{bias2}=42.3\;k\Omega$, where $R_{bias1}$ and $R_{bias2}$ are resistors for the current bias of pin 5 of LT1228-1 and LT1228-2, respectively.

**Figure 15:**
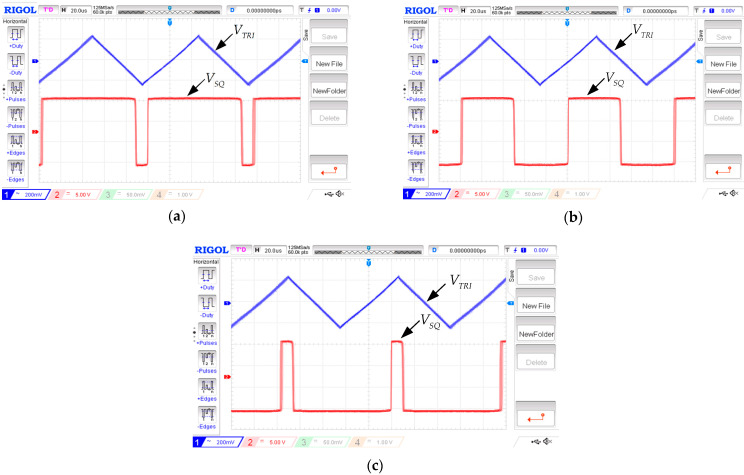
The voltage waveforms with triangular and rectangular wave at a frequency of 10.42 kHz. (**a**) 90% Duty Cycle (**b**) 50% Duty Cycle (**c**) 10% Duty Cycle.

**Figure 16:**
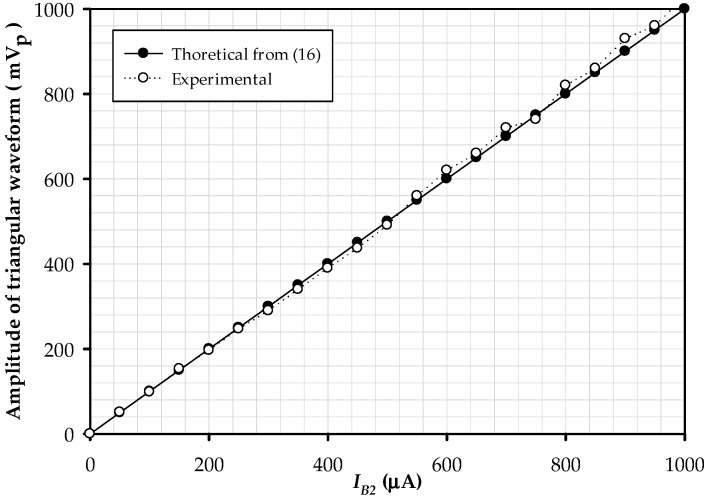
The plot of the magnitude of the triangular signal while adjusting *I*_*B*2_.

Designing the frequency and duty cycles of the proposed circuit are 10 kHz, 90%, 50%, and 10%, which $V_{ref}$ is varied −160 mV, 0 mV, and 160 mV; the amplitude of triangular and square waves, respectively, are 200 ${\mathrm{mV}}_{p}$ and 9 $V_{p}$. Figure 15 displays the triangular and square waves with the frequency of 10.42 kHz when $V_{ref}$ is varied −168 mV, 0 mV, and 168 mV; the duty cycles are 90%, 50%, and 10%; Triangular and square waves have amplitudes of 211 ${\mathrm{mV}}_{p}$ and 7.596 $V_{p}$, respectively. This error is due to the non-ideal case of LT1228, which was discussed in Section 4.3. The results are plotted, as shown in Figure 16; it is the amplitude of the triangular signal, which is controlled by $I_{B2}$.

**Figure 17:**
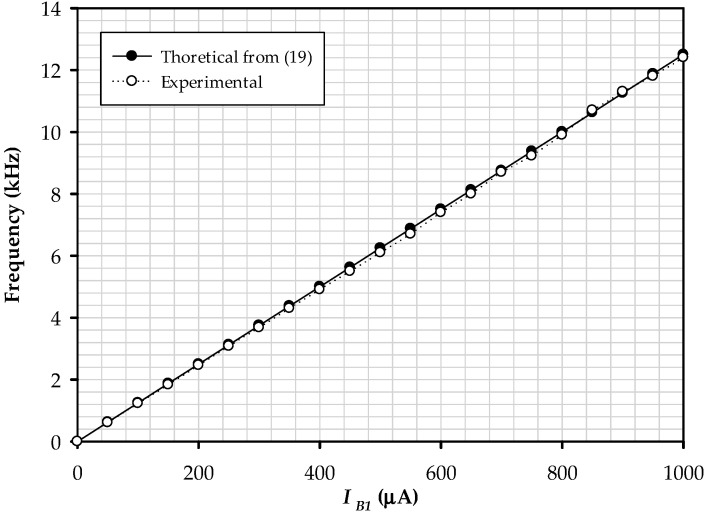
The plot of the frequency when tuning *I*_*B*1_.

Linear controlling the triangular signal’s amplitude is varied in the range of 0–1.03 V when $I_{B2}$ is 0–1 mA. The peak amplitude deviates from the theory analyzed by ±3.33%. The test modulates the frequency by adjusting $I_{B1}$, which found that its dynamics change linearly, as demonstrated in Figure 17.

**Figure 18:**
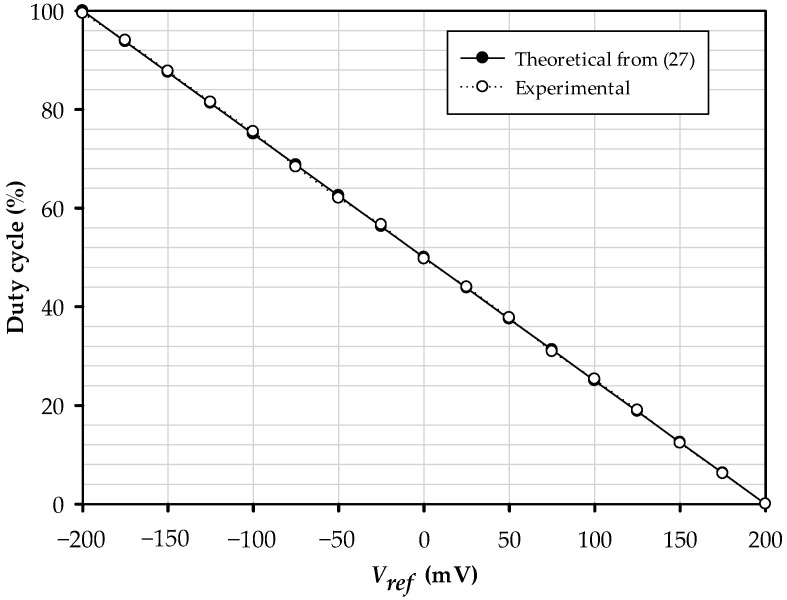
The plot of the rectangular wave’s duty cycle when *V*_*ref*_ variation.

The magnitude of the triangular and square signals is likewise unaffected by frequency modulation. The results obtained have a maximum error of about ±2.55%. The matter of the values of $\alpha_{1}$, $\alpha_{2}$, and $\beta_{x1}$ deviating from one, as shown in (32) The plot is illustrated in Figure 18; it is the comparison between the duty cycle and $V_{ref}$ change from −200 mV to 200 mV. It is confirmed that $V_{ref}$ linearly controls the duty cycle by the electronic technique. The maximum error of the square wave’s duty cycle is ±1.60%.

## 6. Conclusions

Off-the-shelf IC LT1228 designs the proposed triangular/square wave generator for capacitive sensors, which comprises two LT1228s, and a grounded capacitor and resistor. The circuit can be electronically/linearly tuned for the duty cycle, the magnitude triangular wave, and the frequency, which are achieved by $V_{ref}$, $I_{B2}$, and $I_{B1}$, respectively. The amplitude of the triangle signal is not affected by frequency control using $I_{B1}$, which is another advantage of this method. The proposed circuit’s functional test, when $I_{B1}$, $I_{B2}$, R and C are assigned equally to 800 μA, 200 μA, 1 k and 0.1 μF, respectively. The experimental results show that the circuit has a frequency of 10.42 kHz, and when $V_{ref}$ is −168 mV, 0 mV, and 168 mV, the duty cycle is equal to 90%, 50%, and 10%, respectively; the duty cycle is varied by $V_{ref}$, it is changed between −200 mV and 200 mV. The amplitude of the triangular wave can be adjusted from 51 mV to 1.03 V, with $I_{B2}$ in the range from 50 μA to 1 mA. The available frequencies are in the range of 0–12.4 kHz, which $I_{B1}$ is about 0–1 mA. The maximum errors of the duty cycle, triangular wave’s magnitude, and frequency are ±1.60%, ±3.33%, and ±2.55%.

## Data Availability

Not applicable.
